# Association between branched-chain amino acid levels and gastric cancer risk: large-scale prospective cohort study

**DOI:** 10.3389/fnut.2024.1479800

**Published:** 2024-11-20

**Authors:** Liang Yu, Shiming Bao, Feng Zhu, Yanyan Xu, Yanwei Liu, Runben Jiang, Chuang Yang, Feng Cao, Wei Chen, Pengtao Li

**Affiliations:** ^1^Department of General Surgery, The First Affiliated Hospital of Anhui Medical University, Hefei City, China; ^2^Department of General Surgery, The Second Affiliated Hospital of Anhui Medical University, Hefei City, China; ^3^Department of Emergency Surgery, Tongling People's Hospital, Tongling, China; ^4^Department of General Surgery, Tongling People's Hospital, Tongling, China; ^5^Medical Faculty, University of Leipzig, Leipzig, Germany; ^6^Medical Faculty, RWTH Aachen University, Aachen, Germany; ^7^Department of Emergency Medicine, No.2 People's Hospital of Fuyang City, Fuyang City, Anhui Province, China; ^8^Department of Emergency Medicine, Fuyang Infectious Disease Clinical College of Anhui Medical University, Hefei City, China

**Keywords:** amino acid, gastric cancer, UK Biobank, prospective cohort study, cancer prevention

## Abstract

**Background:**

Gastric cancer (GC) remains a malignancy with high incidence and mortality rates worldwide. Although branched-chain amino acids (BCAAs) play a crucial role in various physiological and pathological processes, their specific relationship with risk of GC remains unclear.

**Methods:**

We conducted a large-scale prospective cohort from UK Biobank database. We evaluated the relationship between BCAA levels and risk of GC using Cox regression, Kaplan–Meier survival curves, the accelerated failure time (AFT) model, and restricted cubic spline (RCS) analysis.

**Results:**

During the follow-up of 12 years, 247,753 participants were included in the study. And the Cox regression analysis revealed that higher levels of isoleucine (HR = 0.65, 95% CI 0.48–0.89; *p* = 0.007), leucine (HR = 0.57, 95% CI 0.42–0.79; *p* < 0.001), valine (HR = 0.53, 95% CI 0.39–0.73; *p* < 0.001), and total BCAAs were associated with a reduced risk of GC (HR = 0.51, 95% CI 0.37–0.70; *p* < 0.001). Kaplan–Meier curves and the AFT model confirmed that elevated BCAA levels significantly delayed the onset of GC. Additionally, RCS analysis identified nonlinear dose–response relationships between BCAAs and risk of GC. Stratified analyses indicated that the protective effect of BCAAs was consistent across various subgroups, with a more pronounced impact in older individuals without chronic diseases.

**Conclusion:**

Elevated BCAA levels are significantly associated with a reduced risk of GC, particularly in older adults. This finding highlights the potential of BCAAs in GC prevention and suggests that future research and clinical practice should emphasize regulating BCAA levels.

## Introduction

Gastric cancer (GC) is a prevalent and highly lethal malignancy worldwide. According to the Global Cancer Observatory (GLOBOCAN), >1 million new cases of GC were reported globally in 2022, accounting for 5.6% of all new cancer cases. The mortality rate for GC remains high, with approximately 770,000 deaths annually, making it the third leading cause of cancer-related deaths (1, 2). Many patients are diagnosed at an advanced stage due to the asymptomatic nature of the early disease, leading to poor treatment outcomes and prognosis (3). Despite advancements in early screening and therapeutic approaches, the overall survival rate for GC remains low (4). Therefore, investigating the pathogenesis and risk factors of GC is crucial for identifying effective prevention and treatment strategies.

Amino acids, as fundamental building blocks of proteins, are crucial in sustaining life and regulating bodily functions (5–7). Recent studies have revealed that amino acids are vital not only for metabolic regulation, cellular signaling, and immune responses but also in the onset and progression of various cancers (8, 9). For example, certain amino acids can promote cancer cell growth and proliferation, influence the tumor microenvironment, and serve as potential biomarkers for early cancer detection and prognosis assessment (10, 11). Branched-chain amino acids (BCAAs), including leucine, isoleucine, and valine, are essential amino acids that have garnered increasing attention in research on metabolic diseases and cancer. Despite their recognized roles in other types of cancers, the specific functions and underlying mechanisms of BCCAs in GC remain unclear. Preliminary studies suggest that BCAAs may be involved in the metabolic regulation and proliferation of GC cells, but the detailed mechanisms and effects require further exploration (12). Therefore, a systematic investigation of the relationship between BCAAs and GC is crucial not only to address this research gap but also to potentially uncover new insights and approaches for the prevention and treatment of GC.

Therefore, exploring the correlation between BCAAs and the risk of developing GC holds significant scientific and clinical value. This study, using a large-scale prospective cohort, aims to systematically evaluate the impact of BCAAs on GC risk. The findings will provide a theoretical basis and new research directions for future strategies in the prevention and treatment of GC.

## Methods

### Study population

A total of 502,357 volunteers, aged 37–73 years, were enrolled in UK Biobank (UKB) between 2006 and 2010. Data were collected through a self-administered touchscreen questionnaire, a brief interview, physical and functional measurements, and biological samples collected during the assessment visit. Detailed information has been reported elsewhere (13).

### Measurement of amino acids

Amino acids data were obtained from a nuclear magnetic resonance (NMR) platform, which measured 251 metabolic indicators in >250,000 individuals across two phases: phase 1 (June 2019 to April 2020) and phase 2 (April 2020 to June 2022) (14, 15). This platform provides measurements for nine amino acids, including BCAA. Since the study is based on the data available from the platform, we included all amino acid markers provided to maximize the use of available data for a comprehensive analysis of GC risk. The BCAAs included isoleucine, leucine, and valine. The total BCAA measurement was calculated as the sum of these three amino acids.

### Assessment of GC

Diagnosis of GC was determined using International Classification of Diseases, Tenth Revision (ICD-10) codes C16.0–16.9 from the cancer registry. Data on death cases were obtained from death registries. The follow-up period for participants began on their enrollment date and continued until the earliest of the following events: the first occurrence of GC or another cancer, the participant’s death, or the study cutoff date (June 1, 2022).

### Assessment of other covariates

Researchers collected participants’ demographic and medical information through interviews and touchscreen questionnaires during the baseline visit. This information included age, sex, ethnicity, body mass index (BMI), physical activity levels, food intake frequency, Townsend Deprivation Index (TDI), smoking and drinking habits, history of cardiovascular disease (CVD) and diabetes mellitus (DM), and family history of cancer. Information on the use of lipid-lowering drugs was also recorded. Physical activity was measured in the metabolic equivalent task (MET) minutes (16). The TDI was used to represent socioeconomic status (17). The food intake frequency was used to calculate a diet score ranging from 0 to 9 points, with higher scores indicating poorer dietary habits (18). A family history of cancer was defined as having either parent with a cancer diagnosis. Missing values for baseline covariates were addressed using multiple imputations.

### Selection criteria

Participants with incomplete recruit information and a cancer history at baseline were excluded (*n* = 45,779). Additionally, participants with missing values for any amino acids were excluded (*n* = 208,826). In total, 247,753 participants were included in this study.

### Statistical analysis

BCAA levels were categorized into quartiles (Q1–Q4) to evenly divide the data sample, facilitating a more intuitive observation of how the risk of GC changes with increasing BCAA levels. Specifically, the quartiles were automatically calculated based on the distribution of BCAA levels in the dataset, with Q1 representing the lowest 25% of individuals in terms of BCAA levels, and Q4 representing the highest 25%. This grouping ensures that the number of individuals in each group is approximately equal, thereby reducing potential bias caused by differences in sample size between groups. Descriptive statistics were used to summarize and compare the baseline characteristics of participants across different total BCAA quartiles. Continuous variables were expressed as medians and interquartile ranges, while categorical variables were presented as frequencies and proportions. Group comparisons were performed using ANOVA for continuous variables and the chi-squared test for categorical variables.

To assess the relationship between amino acids and GC risk, multivariable Cox proportional hazard models were used to calculate hazard ratios (HRs) and 95% confidence intervals (CIs). The proportional hazards assumption was verified using Schoenfeld residuals. Amino acid data were categorized into quartiles to examine the effect of quartile increases on GC risk and to test for trends in association. The data were then transformed into z-scores, and HRs were calculated for each 1-unit increase in standard deviation (SD). Two models were established: model 1 adjusted for age, sex, and ethnicity, and model 2 further adjusted for diet score, TDI, MET, BMI, smoking and drinking status, DM, hypertension, CVD, lipid use, and family history of cancer. The cumulative Hazard Kaplan–Meier (KM) Curve was created to assess the hazard of GC across different amino acids quartiles.

Restricted cubic splines (RCS) were used to examine the dose–response relationship between amino acid levels and GC events, with knots placed at the 10th, 50th, and 90th percentiles of amino acid concentrations (19). Subsequently, an accelerated failure time (AFT) model was used to assess the impact of amino acid levels on the timing of GC events. The AFT model, using a Weibull distribution and the lowest quartile (Q1) of amino acids as the reference, evaluated the effects of increased amino acid levels on the acceleration or delay of GC occurrence (20).

To assess potential differences in study outcomes across various population groups, we stratified by age, sex, race, BMI, hypertension, DM, CVD, diet score, family history of cancer, and smoking and alcohol status. We then repeated the primary analyses. Several sensitivity analyses were also conducted to evaluate the robustness of the study findings. First, participants with a follow-up period of <2 years were excluded to mitigate the effect of reverse causality. Second, missing baseline covariate data were removed to assess the impact of imputation on the findings. Third, to account for death as a competing event, the Fine-Gray competing risks model and its sub-distribution were used to describe the impact of amino acid levels on GC risk in the context of competing death states (21). Finally, results from four additional datasets created through multiple imputations were analyzed and summarized to confirm the consistency of the study findings. Statistical analyses were performed using R (v. 4.3.1; R Foundation for Statistical Computing), with statistical significance defined as a two-sided *p* < 0.05.

## Results

### Baseline characteristics

Participants were categorized into quartiles based on their total BCAA levels, revealing significant statistical differences across groups (*p* < 0.05). As BCAA levels increased, the proportion of males and BMI levels increased significantly, while MET levels decreased gradually. Furthermore, the prevalence of diabetes, hypertension, and CVD increased with increasing BCAA levels. Although dietary scores remained consistent across the groups, the proportion of participants using lipid-lowering medications significantly increased with increasing BCAA levels. In terms of smoking and drinking status, the proportion of current smokers slightly decreased as BCAA levels increased, whereas the proportion of former smokers correspondingly increased. These baseline characteristics suggest a significant association between BCAA levels and various demographic and clinical variables, providing crucial background information for further investigation into the relationship between BCAA levels and the risk of developing GC (Table 1).

**Table 1:** Stratified baseline characteristics according to total BCAA quintile levels.

Characteristic	Quintile 1	Quintile 2	Quintile 3	Quintile 4	*p*-Value
	(≤ 0.306)	(0.306–0.354)	(0.354–0.412)	(≥ 0.412)	
Age, years	57.0 (49.0–63.0)	58.0 (50.0–63.0)	58.0 (50.0–63.0)	57.0 (50.0–63.0)	<0.001
Male, *N* (%)	16,393 (26.5%)	26,947 (43.5%)	34,677 (56.0%)	38,648 (62.4%)	<0.001
White, *N* (%)	59,156 (95.5%)	58,828 (95.0%)	58,514 (94.5%)	58,286 (94.1%)	<0.001
BMI	25.1 (22.8–27.9)	26.4 (24.0–29.4)	27.4 (24.9–30.4)	28.2 (25.6–31.4)	<0.001
MET	1893.0 (885.0–3772.0)	1826.0 (836.0–3652.0)	1765.0 (792.0–3519.0)	1693.0 (744.0–3420.0)	<0.001
Townsend deprivation index	−2.1 (−3.6–0.5)	−2.2 (−3.7–0.5)	−2.2 (−3.7–0.4)	−2.2 (−3.7–0.5)	<0.001
Diet score	5.0 (4.0–6.0)	5.0 (4.0–6.0)	5.0 (4.0–6.0)	5.0 (4.0–6.0)	<0.001
DM	1,463 (2.4%)	2,181 (3.5%)	3,405 (5.5%)	5,853 (9.4%)	<0.001
Hypertension	13,602 (22.0%)	16,241 (26.2%)	18,453 (29.8%)	20,330 (32.8%)	<0.001
CVD	3,804 (6.1%)	4,512 (7.3%)	5,198 (8.4%)	6,045 (9.8%)	
History of cancer family	18,441 (29.8%)	18,448 (29.8%)	18,550 (29.9%)	18,499 (29.9%)	0.898
Lipid-lowering drugs, *N* (%)	3,217 (5.2%)	5,490 (8.9%)	7,855 (12.7%)	9,999 (16.1%)	<0.001
Drinking status, *N* (%)					<0.001
Never	2,741 (4.4%)	2,702 (4.4%)	2,669 (4.3%)	2,697 (4.4%)	
Previous	2,298 (3.7%)	2052 (3.3%)	2082 (3.4%)	2,311 (3.7%)	
Current	56,897 (91.9%)	57,186 (92.3%)	57,187 (92.3%)	56,930 (91.9%)	
Smoking status, *N* (%)					<0.001
Never	26,052 (42.1%)	25,139 (40.6%)	24,666 (39.8%)	24,215 (39.1%)	
Previous	28,760 (46.4%)	30,269 (48.9%)	30,971 (50.0%)	31,618 (51.0%)	
Current	7,124 (11.5%)	6,532 (10.5%)	6,301 (10.2%)	6,105 (9.9%)	

BCAA, Branched-chain amino acid; BMI, body mass index; MET, metabolic equivalent task; DM, diabetes mellitus; CVD, cardiovascular diseases.

### Association between BCAAs and GC risk

Cox regression analysis revealed a significant inverse association between higher levels of BCAAs and the risk of GC. In contrast, no such relationship was observed for other amino acids (alanine, glutamine, glycine, histidine, phenylalanine, and tyrosine) (Supplementary Table S1). After adjusting for multiple confounding factors (Model 2), participants in the highest quartile (Q4) of isoleucine, leucine, and valine levels exhibited a 35% (HR 0.65, 95% CI 0.48–0.89; *p* = 0.007), 43% (HR 0.57, 95% CI 0.42–0.79; *p* < 0.001), and 47% (HR 0.53, 95% CI 0.39–0.73; *p* < 0.001) reduction in GC risk, respectively, compared to those in the lowest quartile (Q1). Furthermore, total BCAA levels were also associated with a reduced GC risk (HR 0.51, 95% CI 0.37–0.70; *p* < 0.001) (Table 2). Further analysis revealed that each SD increase in isoleucine, leucine, and valine levels corresponded to a 14% (HR 0.86, 95% CI 0.76–0.97; *p* = 0.012), 15% (HR 0.85, 95% CI 0.75–0.96; *p* = 0.007), and 20% (HR 0.80, 95% CI 0.71–0.91; *p* < 0.001) reduction in GC risk, respectively. Additionally, each SD increase in total BCAA levels was associated with an 18% reduction in GC risk (HR 0.82, 95% CI 0.72–0.93; *p* = 0.002) (Table 2). These results suggest that higher BCAA levels may serve as a significant protective factor against the risk of GC.

**Table 2:** The association between BCAAs and the risk of gastric cancer.

Type	Model 1		Model 2	
	HR (95%CI)	*p*	HR (95%CI)	*p*
Isoleucine				
Q1	Reference		Reference	
Q2	0.82 (0.61–1.1)	0.185	0.79 (0.59–1.07)	0.129
Q3	0.66 (0.49–0.9)	0.009	0.63 (0.46–0.86)	0.003
Q4	0.71 (0.52–0.96)	0.025	0.65 (0.48–0.89)	0.007
*p* for trend	0.013		0.003	
Per SD increase	0.88 (0.78–0.99)	0.035	0.86 (0.76–0.97)	0.012
Leucine				
Q1	Reference		Reference	
Q2	0.7 (0.52–0.96)	0.027	0.69 (0.51–0.95)	0.021
Q3	0.77 (0.57–1.03)	0.081	0.74 (0.55–1)	0.052
Q4	0.61 (0.44–0.83)	0.002	0.57 (0.42–0.79)	<0.001
*p* for trend	0.007		0.002	
Per SD increase	0.87 (0.77–0.97)	0.017	0.85 (0.75–0.96)	0.007
Valine				
Q1	Reference		Reference	
Q2	0.67 (0.5–0.91)	0.01	0.65 (0.48–0.89)	0.007
Q3	0.68 (0.51–0.92)	0.012	0.65 (0.48–0.88)	0.005
Q4	0.59 (0.43–0.8)	<0.001	0.53 (0.39–0.73)	<0.001
*p* for trend	0.002		< 0.001	
Per SD increase	0.84 (0.74–0.94)	0.003	0.8 (0.71–0.91)	<0.001
Total BCAA				
Q1	Reference		Reference	
Q2	0.63 (0.47–0.86)	0.003	0.62 (0.45–0.83)	0.002
Q3	0.63 (0.47–0.85)	0.003	0.6 (0.44–0.81)	<0.001
Q4	0.56 (0.41–0.76)	<0.001	0.51 (0.37–0.7)	<0.001
*p* for trend	< 0.001		< 0.001	
Per SD increase	0.85 (0.75–0.95)	0.007	0.82 (0.72–0.93)	0.002

BCAA, Branched-chain amino acid; GC, gastric cancer; HR, hazard ratio; CI, confidence interval; SD, standard deviation; Model 1 adjusted for age, sex, and ethnicity; Model 2 further adjusted for diet score, TDI, MET, BMI, smoking and drinking status, DM, Hypertension, CVD, lipid, and family history of cancer.

### Association between BCAAs and cumulative GC risk

**Figure 1:**
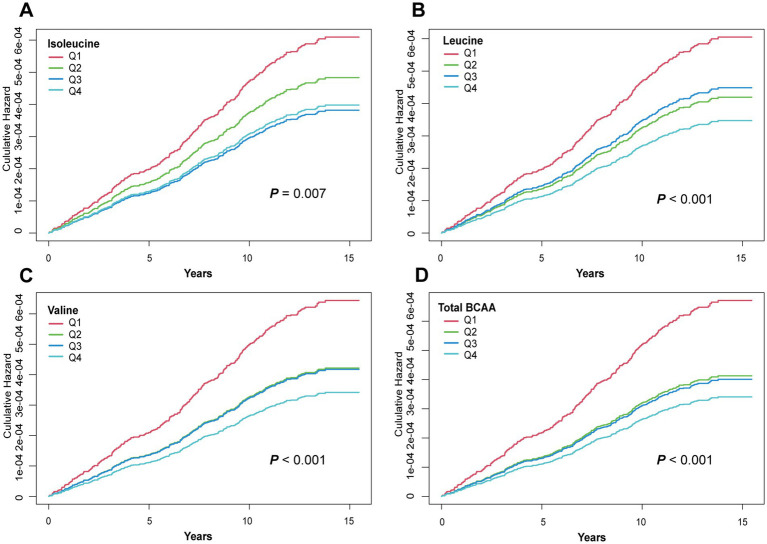
Kaplan–Meier curves for GC events in the BCAAs group. **(A)** Isoleucine. **(B)** Leucine. **(C)** Valine. **(D)** Total BCAA. Models were adjusted with age, sex, and ethnicity, BMI (body mass index), MET (metabolic equivalent task), TDI (Townsend Deprivation Index), smoking and drinking status, DM (diabetes mellitus) Hypertension, CVD, lipid, and family history of cancer. BCAA: Branched-chain amino acid.

To further validate the association between BCAA levels and GC risk, KM survival curves were used to compare the cumulative risk across different BCAA quintiles. As shown in Figure 1, the cumulative risk of GC in the highest BCAA level group (Q4) for leucine, valine, and total BCAAs was significantly lower than in the lowest BCAA level group (Q1), with statistically significant differences (leucine *p* < 0.001, valine *p* < 0.001, and total BCAA *p* < 0.001). Moreover, the cumulative risk for the Q4 group remained consistently lower than that for the Q1 group over the follow-up period (Figure 1). Surprisingly, the lowest cumulative risk for isoleucine was observed in the Q3 group (*p* = 0.007). These findings further support the role of BCAAs as potential protective factors in reducing GC risk, demonstrating a clear trend where higher BCAA levels are associated with lower GC risk.

### Dose–response relationships between BCAAs and GC

**Figure 2:**
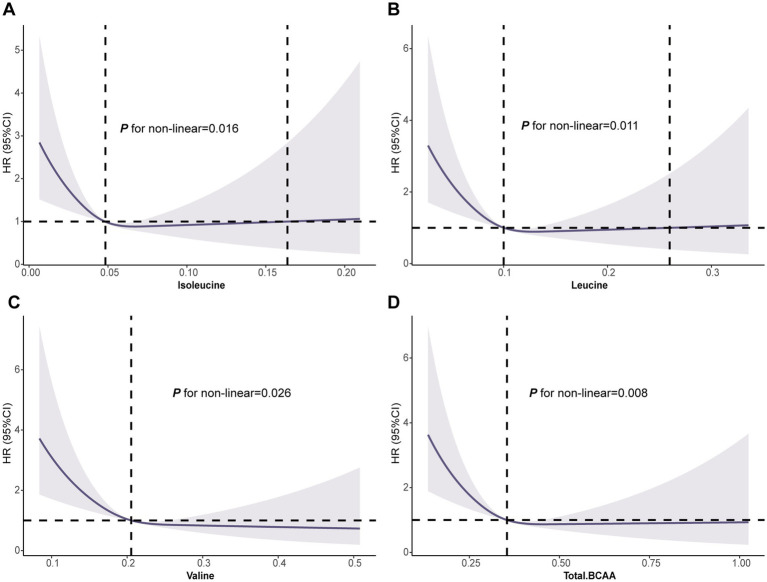
Association of the BCAAs with GC using RCS. **(A)** Isoleucine. **(B)** Leucine. **(C)** Valine. **(D)** Total BCAA. Models were adjusted with age, sex, and ethnicity, BMI (body mass index), MET (metabolic equivalent task), TDI (Townsend Deprivation Index), smoking and drinking status, DM (diabetes mellitus) Hypertension, CVD, lipid, and family history of cancer. BCAA: Branched-chain amino acid.

The RCS analysis revealed a significant nonlinear association between BCAA levels and GC risk (leucine *p* for nonlinearity = 0.011, valine *p* for nonlinearity = 0.026, and total BCAA *p* for nonlinearity = 0.008). At lower BCAA levels, the risk of GC was higher. As BCAA levels increased, the risk of GC gradually decreased, with a marked reduction in GC incidence at higher BCAA levels (Figure 2). Interestingly, for isoleucine, there was an observed increase in GC risk at excessively high levels (isoleucine *p* for non-linearity = 0.016) (Figure 2). This nonlinear dose–response relationship highlights the potential protective role of BCAAs in GC prevention and suggests that BCAAs may offer optimal protective effects within a specific range. These findings provide valuable insights for exploring the mechanisms and determining the optimal dosages of BCAAs for GC prevention and treatment.

### AFT analysis of BCAA levels and GC risk

**Figure 3:**
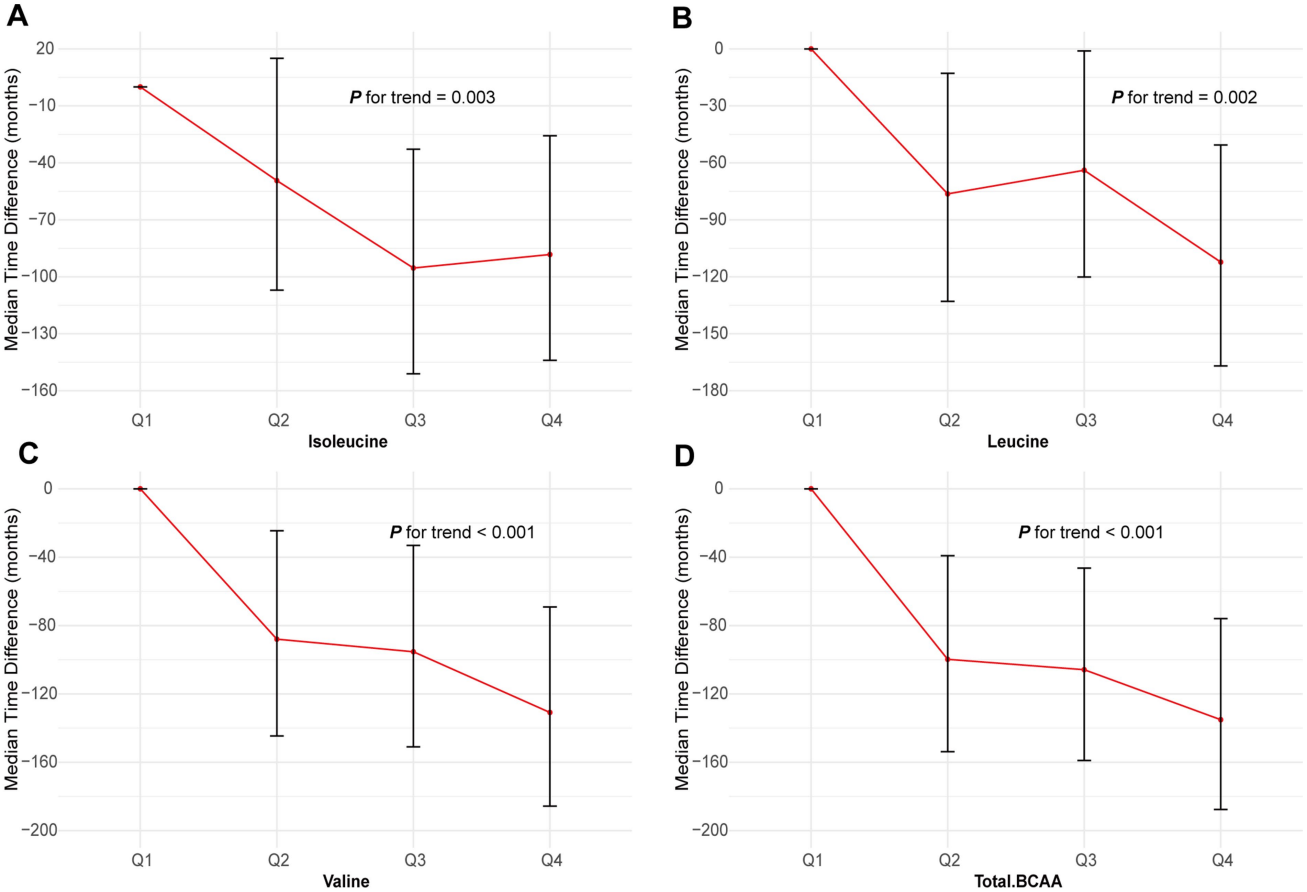
Association of the BCAAs with GC using AFT. **(A)** Isoleucine. **(B)** Leucine. **(C)** Valine. **(D)** Total BCAA. Models were adjusted with age, sex, and ethnicity, BMI (body mass index), MET (metabolic equivalent task), TDI (Townsend Deprivation Index), smoking and drinking status, DM (diabetes mellitus) Hypertension, CVD, lipid, and family history of cancer. BCAA: Branched-chain amino acid.

The AFT model demonstrated that higher levels of BCAAs significantly delayed the onset of GC. However, it was observed that the average time to GC onset for participants in the highest quartile (Q4) of isoleucine occurred earlier compared to those in the third quartile (Q3) (Figure 3). Furthermore, compared to the lowest quartile (Q1), the median time differences for isoleucine in the Q2, Q3, and Q4 quartiles were 2.75 months, 21.49 months, and 68.71 months, respectively (*p* = 0.036). For leucine, valine, and total BCAA, the median time differences in the Q4 quartile compared to Q1 were 88.28 months, 112.27 months, and 130.89 months, respectively (leucine *p* = 0.003, valine *p* = 0.002, and total BCAA *p* < 0.001) (Supplementary Table S2). These results underscore the protective role of higher BCAA levels in delaying the onset of GC.

### Sensitivity analysis

To validate the robustness of the Cox regression results, we conducted three sensitivity analyses and a Fine-Gray competing risk model analysis. After excluding participants who developed GC within 2 years, higher levels of isoleucine (HR 0.65, 95% CI 0.47–0.91; *p* = 0.012), leucine (HR 0.58, 95% CI 0.41–0.81; *p* = 0.002), valine (HR 0.51, 95% CI 0.36–0.72; *p* < 0.001), and total BCAAs (HR 0.51, 95% CI 0.36–0.72; *p* < 0.001) remained significantly associated with a reduced risk of GC (Supplementary Table S3). Trend analyses for all BCAAs showed significant *p*-values (isoleucine *p* = 0.008, leucine *p* = 0.006, valine *p* < 0.001, and total BCAA *p* < 0.001) (Supplementary Table S3). Furthermore, analyses excluding participants with missing baseline covariates and those using multiple imputation techniques consistently supported these conclusions, indicating that higher BCAA levels still significantly reduced GC risk. The risk ratios per SD increase further emphasized the significant protective effect of BCAAs (Supplementary Tables S4, S5). Moreover, the Fine-Gray competing risk model indicated that compared to the lowest quartile (Q1), the highest quartile (Q4) of isoleucine, leucine, valine, and total BCAA levels were associated with reductions in GC risk of 34, 41, 46, and 48%, respectively (Supplementary Table S6). These results are consistent with the Cox regression analysis and reinforce the reliability of the study’s conclusions regarding the impact of BCCAs on GC risk.

### Subgroup analysis of BCAA levels and GC risk

**Figure 4:**
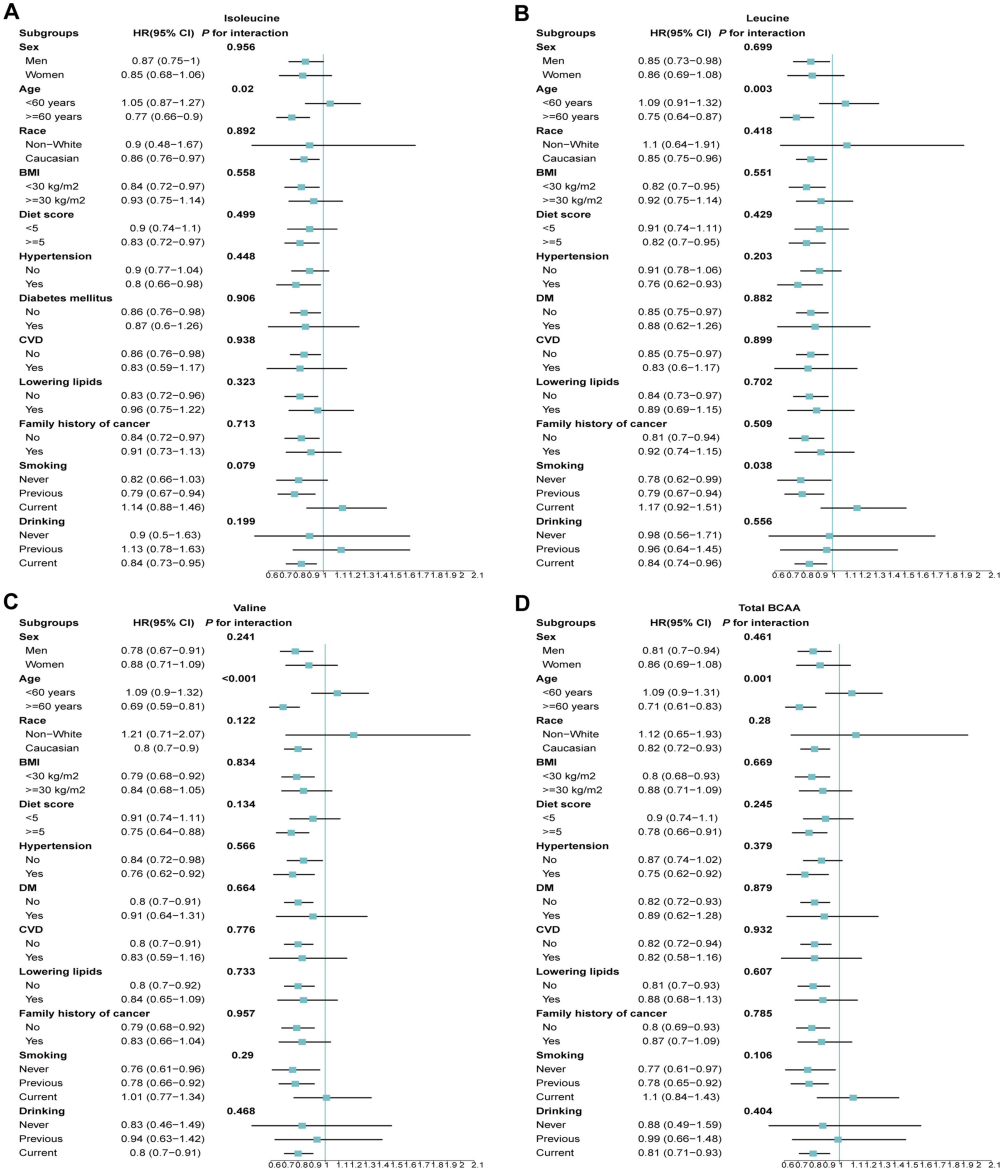
The association between BCAAs and the risk of GC across different demographic and clinical subgroups. **(A)** Isoleucine. **(B)** Leucine. **(C)** Valine. **(D)** Total BCAA. Models were adjusted with age, sex, and ethnicity, BMI (body mass index), MET (metabolic equivalent task), TDI (Townsend Deprivation Index), smoking and drinking status, DM (diabetes mellitus) Hypertension, CVD, lipid, and family history of cancer. BCAA: Branched-chain amino acid.

To further investigate the protective effects of BCAA levels across different demographic and clinical subgroups, we conducted stratified analyses. The results revealed that isoleucine, leucine, valine, and total BCAA levels did not exhibit significant interactions with GC risk across various subgroups based on gender, BMI, smoking status, drinking status, or the presence of diabetes and hypertension (Figure 4). However, significant heterogeneity was observed when stratifying by age. Higher BCAA levels were associated with a reduced risk of GC in participants older than 60 years, while the risk slightly increased in those younger than 60 years. Additionally, an interaction was observed for leucine concerning smoking status. Specifically, current smokers showed a 17% increased risk of GC, whereas the risk was significantly reduced among never smokers and former smokers (Figure 4).

## Discussion

This study provides a comprehensive analysis of the effects of BCAAs on the risk of GC. The findings demonstrate that higher levels of isoleucine, leucine, valine, and total BCAAs are significantly associated with a reduced risk of GC. Stratified analyses further reveal that age influences the relationship between BCAAs and GC risk, with the protective effects of BCAAs being particularly pronounced in older individuals. Additionally, elevated BCAA levels were found to significantly delay the onset of GC. These results offer novel scientific insights into GC prevention strategies and highlight the potential role of BCAAs in oncology. They suggest that modulation of BCAA levels could be a valuable approach for preventing and delaying the onset of GC.

Previous research has reported similar protective effects of BCAAs across various cancer types. For example, some studies have reported an association between high levels of BCAAs and a reduced risk of liver cancer (22). Moreover, BCAAs may help decrease the incidence of obesity-related cancers by modulating metabolic pathways and improving insulin sensitivity (23, 24). Research by Viana et al. (25) suggested that higher levels of leucine could shift tumor metabolism from glycolysis to oxidative phosphorylation components, leading to increased mRNA and protein expression of oxidative phosphorylation components and consequently reduced tumor invasiveness and metastasis. These findings are consistent with our results and support the potential protective role of BCAAs in lowering cancer risk. However, an alternative perspective is provided by Tang et al. (26), who reported that leucine and arginine might promote colorectal cancer cell proliferation by activating the PI3K/Akt/Wnt/*β*-catenin pathway. This discrepancy may be attributed to the use of microcystins (MCs) in their study. MCs are potent carcinogens produced by cyanobacteria and are known for their strong carcinogenic properties (27–29). Although the PI3K/Akt/Wnt/β-catenin pathway is crucial in cell proliferation and cancer development, its activation and regulation are highly complex and influenced by various internal and external factors. Therefore, we propose that the complex role of BCAAs in different cancer types may be influenced by factors such as cancer type, tumor microenvironment, and the metabolic state of the patient. Several physiological and molecular pathways could be involved in the mechanisms by which BCAAs regulate GC risk. First, BCAAs, particularly leucine, modulate protein synthesis and cell growth through the activation of the mTOR (mammalian target of rapamycin) signaling pathway (30). mTOR is a crucial regulator involved in tumor cell growth, proliferation, and survival (31). While overactive mTOR signaling is associated with many cancer types, moderate mTOR activity may help maintain normal cell functions and prevent excessive proliferation of cancer cells (32). Second, BCAAs play a significant role in regulating energy metabolism. By enhancing insulin sensitivity and improving glucose metabolism, BCAAs help prevent obesity and diabetes, which are major risk factors for GC (24, 33). Additionally, BCAAs have anti-inflammatory properties that can reduce chronic inflammatory responses in the body (34). Chronic inflammation is a key factor in cancer development and progression, thus reducing inflammation through BCAAs may indirectly lower cancer risk. Furthermore, BCAAs may also influence the regulation of the gut microbiota. Dysbiosis, or imbalance in the gut microbiota, is closely linked with various diseases, including cancer (35). Studies have shown that BCAAs can modulate gut microbiota composition, enhance gut barrier function, and reduce the proliferation of harmful bacteria, potentially contributing to a decreased risk of GC (36). Furthermore, it is noteworthy that the mechanisms through which BCAAs exert their effects may extend beyond direct influences on cell growth and metabolism to include the modulation of the host’s immune system. Research indicates that BCAAs may play a role in antitumor immune responses by affecting immune cell function. For instance, BCAAs can influence the metabolic pathways of T cells, thereby promoting their function and proliferation and enhancing the body’s immune surveillance against tumors (37). Additionally, BCAAs may regulate the activity of immunosuppressive cells, such as modulating the function of regulatory T cells (Tregs) and reducing their inhibitory effects on antitumor responses (38). These mechanisms may further elucidate the protective effects of BCAAs in reducing cancer risk.

In clinical practice, the supplementation or modulation of BCAAs could have considerable practical implications. Given the metabolic characteristics of BCAAs and their potential protective role in GC, developing personalized BCAA intervention strategies is crucial. These strategies should be tailored based on the patient’s disease stage, metabolic status, and genetic background. For instance, in certain patients with GC, especially those with abnormal BCAA levels or disrupted metabolism, adjusting BCAA levels might be necessary to support and enhance treatment efficacy. Conversely, in individuals without cancer or those at high risk, increasing BCAA intake may help reduce the risk of GC, thereby serving a preventive role. To achieve these potential clinical applications, further clinical trials are essential to validate the safety and efficacy of BCAA interventions and to determine the optimal intervention protocols and dosages. These studies should account for individual differences, including genomic information, metabolic state, and lifestyle factors, to ensure that interventions are tailored to the specific needs of diverse populations.

This study’s strengths include its large-scale prospective design and long-term follow-up data, which enhance the statistical significance and robustness of the results. Accurate NMR measurements of BCAA levels help mitigate recall bias from dietary questionnaires, thus strengthening the reliability of the conclusions. However, despite providing strong evidence regarding the relationship between BCAAs and GC risk, several limitations must be acknowledged. First, as an observational study, it cannot establish causality. Second, BCAA levels were measured only at baseline, precluding the assessment of their effects over time and how fluctuations in these levels might influence cancer risk. Third, the study population primarily consists of individuals from a specific region in the UK, which may limit the generalizability of the findings to other populations, particularly those from different ethnic or geographic backgrounds. Finally, despite adjusting for multiple confounders, some unmeasured variables, such as genetic background, gut microbiota composition, or specific dietary patterns, may still influence the results. Future research should employ longitudinal designs, include more diverse sample populations, and explore the specific mechanisms through which BCAAs affect different groups to better understand their role in cancer prevention.

In conclusion, this study found that higher levels of BCAAs are significantly associated with a reduced risk of GC, with particularly pronounced protective effects in older individuals and those without chronic diseases. These findings provide preliminary evidence for the potential use of BCAAs in the prevention and treatment of GC. However, further research is needed to elucidate the specific mechanisms and to determine the optimal methods for their use. Such research will facilitate the development of more personalized and effective intervention strategies for patients with cancer and high-risk populations, thereby advancing the field of GC prevention and treatment.

## Data Availability

The raw data supporting the conclusions of this article will be made available by the authors, without undue reservation.

## References

[1] Bray F Laversanne M Sung H Ferlay J Siegel RL Soerjomataram I. Global cancer statistics 2022: GLOBOCAN estimates of incidence and mortality worldwide for 36 cancers in 185 countries. CA Cancer J Clin. (2024) 74:229–63. doi: 10.3322/caac.21834, PMID: 38572751

[2] Siegel RL Wagle NS Cercek A Smith RA Jemal A. Colorectal cancer statistics, 2023. CA Cancer J Clin. (2023) 73:233–54. doi: 10.3322/caac.21772, PMID: 36856579

[3] Thommen DS Schumacher TN. T cell dysfunction in Cancer. Cancer Cell. (2018) 33:547–62. doi: 10.1016/j.ccell.2018.03.012, PMID: 29634943 PMC7116508

[4] Zhang XY Zhang PY. Gastric cancer: somatic genetics as a guide to therapy. J Med Genet. (2017) 54:305–12. doi: 10.1136/jmedgenet-2016-104171, PMID: 27609016

[5] Paulusma CC Lamers WH Broer S van de Graaf SFJ. Amino acid metabolism, transport and signalling in the liver revisited. Biochem Pharmacol. (2022) 201:115074. doi: 10.1016/j.bcp.2022.115074, PMID: 35568239

[6] Broer S. Amino acid transporters as modulators of glucose homeostasis. Trends Endocrinol Metab. (2022) 33:120–35. doi: 10.1016/j.tem.2021.11.004, PMID: 34924221

[7] Flynn NE Shaw MH Becker JT. Amino acids in health and endocrine function. Adv Exp Med Biol. (2020) 1265:97–109. doi: 10.1007/978-3-030-45328-2_6, PMID: 32761572

[8] Li P Yin YL Li D Kim SW Wu G. Amino acids and immune function. Br J Nutr. (2007) 98:237–52. doi: 10.1017/S000711450769936X 17403271

[9] Saito Y Soga T. Amino acid transporters as emerging therapeutic targets in cancer. Cancer Sci. (2021) 112:2958–65. doi: 10.1111/cas.15006, PMID: 34091991 PMC8353895

[10] Peng H Wang Y Luo W. Multifaceted role of branched-chain amino acid metabolism in cancer. Oncogene. (2020) 39:6747–56. doi: 10.1038/s41388-020-01480-z, PMID: 32978521 PMC7606751

[11] Bi X Henry CJ. Plasma-free amino acid profiles are predictors of cancer and diabetes development. Nutr Diabetes. (2017) 7:e249. doi: 10.1038/nutd.2016.55, PMID: 28287627 PMC5380892

[12] Qian L Li N Lu XC Xu M Liu Y Li K. Enhanced BCAT1 activity and BCAA metabolism promotes rho C activity in cancer progression. Nat Metab. (2023) 5:1159–73. doi: 10.1038/s42255-023-00818-7, PMID: 37337119

[13] Sudlow C Gallacher J Allen N Beral V Burton P Danesh J. UK biobank: an open access resource for identifying the causes of a wide range of complex diseases of middle and old age. PLoS Med. (2015) 12:e1001779. doi: 10.1371/journal.pmed.1001779, PMID: 25826379 PMC4380465

[14] Wurtz P Kangas AJ Soininen P Lawlor DA Davey Smith G Ala-Korpela M. Quantitative serum nuclear magnetic resonance metabolomics in large-scale epidemiology: a primer on-Omic technologies. Am J Epidemiol. (2017) 186:1084–96. doi: 10.1093/aje/kwx016, PMID: 29106475 PMC5860146

[15] Liu Z Huang H Xie J Xu Y Xu C. Circulating fatty acids and risk of hepatocellular carcinoma and chronic liver disease mortality in the UK biobank. Nat Commun. (2024) 15:3707. doi: 10.1038/s41467-024-47960-8, PMID: 38697980 PMC11065883

[16] Chudasama YV Khunti KK Zaccardi F Rowlands AV Yates T Gillies CL. Physical activity, multimorbidity, and life expectancy: a UK biobank longitudinal study. BMC Med. (2019) 17:108. doi: 10.1186/s12916-019-1339-0, PMID: 31186007 PMC6560907

[17] Ye J Wen Y Sun X Chu X Li P Cheng B. Socioeconomic deprivation index is associated with psychiatric disorders: an observational and genome-wide gene-by-environment interaction analysis in the UK biobank cohort. Biol Psychiatry. (2021) 89:888–95. doi: 10.1016/j.biopsych.2020.11.019, PMID: 33500177

[18] Petermann-Rocha F Ho FK Foster H Boopor J Parra-Soto S Gray SR. Nonlinear associations between cumulative dietary risk factors and cardiovascular diseases, Cancer, and all-cause mortality: a prospective cohort study from UK biobank. Mayo Clin Proc. (2021) 96:2418–31. doi: 10.1016/j.mayocp.2021.01.036, PMID: 34366141

[19] Arnes JI Hapfelmeier A Horsch A Braaten T. Greedy knot selection algorithm for restricted cubic spline regression. Front Epidemiol. (2023) 3:1283705. doi: 10.3389/fepid.2023.1283705, PMID: 38455941 PMC10910934

[20] Su S. Flexible parametric accelerated failure time model. J Biopharm Stat. (2021) 31:650–67. doi: 10.1080/10543406.2021.1934854, PMID: 34550051

[21] Austin PC Fine JP. Practical recommendations for reporting Fine-Gray model analyses for competing risk data. Stat Med. (2017) 36:4391–400. doi: 10.1002/sim.7501, PMID: 28913837 PMC5698744

[22] Liu Y Wang F Yan G Tong Y Guo W Li S. CPT1A loss disrupts BCAA metabolism to confer therapeutic vulnerability in TP53-mutated liver cancer. Cancer Lett. (2024) 595:217006. doi: 10.1016/j.canlet.2024.217006, PMID: 38823763

[23] Yoneshiro T Wang Q Tajima K Matsushita M Maki H Igarashi K. BCAA catabolism in brown fat controls energy homeostasis through SLC25A44. Nature. (2019) 572:614–9. doi: 10.1038/s41586-019-1503-x, PMID: 31435015 PMC6715529

[24] Gannon NP Schnuck JK Vaughan RA. BCAA metabolism and insulin sensitivity-dysregulated by metabolic status? Mol Nutr Food Res. (2018) 62:e1700756. doi: 10.1002/mnfr.201700756, PMID: 29377510

[25] Viana LR Tobar N Busanello ENB Marques AC de Oliveira AG Lima TI. Leucine-rich diet induces a shift in tumour metabolism from glycolytic towards oxidative phosphorylation, reducing glucose consumption and metastasis in Walker-256 tumour-bearing rats. Sci Rep. (2019) 9:15529. doi: 10.1038/s41598-019-52112-w, PMID: 31664147 PMC6820796

[26] Tang Y Yi X Zhang X Liu B Lu Y Pan Z. Microcystin-leucine arginine promotes colorectal cancer cell proliferation by activating the PI3K/Akt/Wnt/beta-catenin pathway. Oncol Rep. (2023) 49:18. doi: 10.3892/or.2022.8455 36453240 PMC9773010

[27] Liu H Guo X Liu L Yan M Li J Hou S. Simultaneous microcystin degradation and *Microcystis aeruginosa* inhibition with the single enzyme microcystinase A. Environ Sci Technol. (2020) 54:8811–20. doi: 10.1021/acs.est.0c02155, PMID: 32463659

[28] Xiao C Mei F Ren G Long L Chen M Fang X. Synergistic effect of MC-LR and C-terminal truncated HBx on Hep G2 cells and their effects on PP2A mediated downstream target of MAPK signaling pathway. Front Genet. (2020) 11:537785. doi: 10.3389/fgene.2020.537785, PMID: 33193609 PMC7593820

[29] Fujiki H Suganuma M. Tumor promoters – microcystin-LR, nodularin and TNF-α and human Cancer development. Anti Cancer Agents Med Chem. (2011) 11:4–18. doi: 10.2174/187152011794941163, PMID: 21269254

[30] Ananieva EA Powell JD Hutson SM. Leucine metabolism in T cell activation: mTOR signaling and beyond. Adv Nutr. (2016) 7:798S–805S. doi: 10.3945/an.115.011221, PMID: 27422517 PMC4942864

[31] Zhang L Han J. Branched-chain amino acid transaminase 1 (BCAT1) promotes the growth of breast cancer cells through improving mTOR-mediated mitochondrial biogenesis and function. Biochem Biophys Res Commun. (2017) 486:224–31. doi: 10.1016/j.bbrc.2017.02.101, PMID: 28235484

[32] Huang S. mTOR signaling in metabolism and cancer. Cells. (2020) 9:2278. doi: 10.3390/cells9102278, PMID: 33065976 PMC7601420

[33] De Bandt JP Coumoul X Barouki R. Branched-chain amino acids and insulin resistance, from protein supply to diet-induced obesity. Nutrients. (2022) 15:68. doi: 10.3390/nu15010068, PMID: 36615726 PMC9824001

[34] Kittithaworn AA Dogra P Saini J Gruppen EG Atkinson E Achenbach S. Enhanced chronic inflammation and increased branched chain amino acids in adrenal disorders: a cross-sectional study. J Clin Endocrinol Metab. (2024). [ahead of Print]. doi: 10.1210/clinem/dgae204, PMID: 38546526 PMC11747673

[35] Gojda J Cahova M. Gut microbiota as the link between elevated BCAA serum levels and insulin resistance. Biomol Ther. (2021) 11:1414. doi: 10.3390/biom11101414, PMID: 34680047 PMC8533624

[36] Chen J Liu X Zou Y Gong J Ge Z Lin X. A high-fat diet promotes cancer progression by inducing gut microbiota-mediated leucine production and PMN-MDSC differentiation. Proc Natl Acad Sci USA. (2024) 121:e2306776121. doi: 10.1073/pnas.2306776121, PMID: 38709933 PMC11098111

[37] Yao CC Sun RM Yang Y Zhou HY Meng ZW Chi R. Accumulation of branched-chain amino acids reprograms glucose metabolism in CD8(+) T cells with enhanced effector function and anti-tumor response. Cell Rep. (2023) 42:112186. doi: 10.1016/j.celrep.2023.112186, PMID: 36870057

[38] Ikeda K Kinoshita M Kayama H Nagamori S Kongpracha P Umemoto E. Slc 3a2 mediates branched-chain amino-acid-dependent maintenance of regulatory T cells. Cell Rep. (2017) 21:1824–38. doi: 10.1016/j.celrep.2017.10.082, PMID: 29141216

