# Supplementary material for: Association between branched-chain amino acid levels and gastric cancer risk: large-scale prospective cohort study
Source: Front Nutr. 2024 Nov 20;11:1479800. doi: 10.3389/fnut.2024.1479800 (PMC11614650; doi:10.3389/fnut.2024.1479800)
Supplement: Supplementary file 1 [file Table_1.DOCX]

Association Between Branched-Chain Amino Acid Levels and Gastric Cancer Risk: Large-Scale Prospective Cohort Study

**Liang Yu^1,2†^, Shiming Bao^3†^, Feng Zhu^4†^, Yanyan Xu^2^, Yanwei Liu^2^, Runben Jiang^2^, Chuang Yang^5^, Feng Cao^6^, Wei Chen^1^*, Pengtao Li^7^***

^1^Department of General Surgery, The First Affiliated Hospital of Anhui Medical University, Hefei City, Anhui Province, China

^2^Department of General Surgery, The Second Affiliated Hospital of Anhui Medical University

^3^Department of Emergency Surgery, Tongling People's Hospital

^4^Department of General Surgery, Tongling People's Hospital

^5^Medical Faculty, University of Leipzig, 04103, Leipzig, Germany.

^6^Medical Faculty, RWTH Aachen university, 52074, Aachen, Germany.

^7^Department of Emergency Medcine, N0.2 People's Hospital of Fuyang City, Fuyang City, Anhui Province, China

| Table S1. The association between other amino acids and the risk of GC | | | | |
| --- | --- | --- | --- | --- |
| Type | Model 1 |  | Model 2 |  |
|  | HR (95%CI) | *P* | HR (95%CI) | *P* |
| Alanine | 0.89 (0.8-1.02) | 0.076 | 0.88 (0.79-1.00) | 0.052 |
| Glutamine | 0.95 (0.85-1.06) | 0.361 | 0.97 (0.87-1.08) | 0.582 |
| Glycine | 1.02 (0.9-1.16) | 0.785 | 1.05 (0.92-1.19) | 0.489 |
| Histidine | 0.99 (0.88-1.11) | 0.847 | 1 (0.9-1.12) | 0.969 |
| Phenylalanine | 0.98 (0.87-1.1) | 0.696 | 0.96 (0.85-1.08) | 0.485 |
| Tyrosine | 0.92 (0.82-1.03) | 0.15 | 0.9 (0.81-1.01) | 0.0838 |

GC: gastric cancer; HR: hazard ratio; CI: confidence interval; SD: standard deviation; Model 1 adjusted for age, sex, and ethnicity; Model 2 further adjusted for diet score, TDI, MET, BMI, smoking and drinking status, DM, Hypertension, CVD, lipid, and family history of cancer.

| Table S2. Adjusted median time difference (month) for GC occurrence in the second, third and fourth quintile groups compared to the BCAAs lowest quintile (Q1) groups. | | | | | | | | | | | |
| --- | --- | --- | --- | --- | --- | --- | --- | --- | --- | --- | --- |
| Type | Q1 | Q2 | | | Q3 | | | Q4 | | |  |
|  |  | median | 95% CI low | 95% CI high | median | 95% CI low | 95% CI high | median | 95% CI low | 95% CI high | *P* for trend |
| Isoleucine | Reference | 2.75 | 74.4 | -61.48 | -21.49 | 48.83 | -84.38 | -68.71 | -0.01 | -129.76 | 0.036 |
| Leucine | Reference | -49.35 | 15.08 | -107.06 | -95.39 | -32.84 | -151.06 | -88.28 | -25.74 | -144.03 | 0.003 |
| Valine | Reference | -76.34 | -12.85 | -132.93 | -63.9 | -1.06 | -120.11 | -112.27 | -50.59 | -166.98 | 0.002 |
| Total BCAA | Reference | -88 | -24.56 | -144.66 | -95.36 | -33.12 | -150.99 | -130.89 | -69.12 | -185.71 | <0.001 |

BCAA: Branched-chain amino acid; GC: gastric cancer; HR: hazard ratio; CI: confidence interval; SD: standard deviation; Models were fully adjusted for age, sex, ethnicity, diet score, TDI, MET, BMI, smoking and drinking status, DM, Hypertension, CVD, lipid, and family history of cancer.

| **Table S3. The association between BCAAs and the risk of GC after excluding participants with follow-up within 2 years** | | |
| --- | --- | --- |
| Type | HR (95%CI) | *P* |
| Isoleucine |  |  |
| Q1 | Reference |  |
| Q2 | 0.79 (0.57-1.09) | 0.154 |
| Q3 | 0.65 (0.46-0.91) | 0.011 |
| Q4 | 0.65 (0.47-0.91) | 0.012 |
| *P* for trend | 0.008 |  |
| Per SD increase | 0.85 (0.74-0.97) | 0.013 |
| Leucine |  |  |
| Q1 | Reference |  |
| Q2 | 0.68 (0.48-0.95) | 0.022 |
| Q3 | 0.74 (0.53-1.02) | 0.067 |
| Q4 | 0.58 (0.41-0.81) | 0.002 |
| *P* for trend | 0.006 |  |
| Per SD increase | 0.85 (0.75-0.97) | 0.018 |
| Valine |  |  |
| Q1 | Reference |  |
| Q2 | 0.69 (0.5-0.96) | 0.025 |
| Q3 | 0.65 (0.47-0.9) | 0.01 |
| Q4 | 0.51 (0.36-0.72) | <0.001 |
| *P* for trend | < 0.001 |  |
| Per SD increase | 0.79 (0.69-0.8) | <0.001 |
| Total BCAA |  |  |
| Q1 | Reference |  |
| Q2 | 0.65 (0.47-0.9) | 0.01 |
| Q3 | 0.6 (0.43-0.83) | 0.002 |
| Q4 | 0.51 (0.36-0.72) | <0.001 |
| *P* for trend | < 0.001 |  |
| Per SD increase | 0.81 (0.71-0.93) | 0.002 |

BCAA: Branched-chain amino acid; GC: gastric cancer; HR: hazard ratio; CI: confidence interval; SD: standard deviation; Models were fully adjusted for age, sex, ethnicity, diet score, TDI, MET, BMI, smoking and drinking status, DM, Hypertension, CVD, lipid, and family history of cancer.

| **Table S4. The association between circulating fatty acids and the risk of GC after excluding participants with any missing value at baseline** | | |
| --- | --- | --- |
| Type | HR (95%CI) | *P* |
| Isoleucine |  |  |
| Q1 | Reference |  |
| Q2 | 0.78 (0.55-1.09) | 0.14 |
| Q3 | 0.61 (0.43-0.87) | 0.006 |
| Q4 | 0.59 (0.41-0.84) | 0.003 |
| *P* for trend | 0.002 |  |
| Per SD increase | 0.84 (0.73-0.97) | 0.016 |
| Leucine |  |  |
| Q1 | Reference |  |
| Q2 | 0.79 (0.56-1.12) | 0.185 |
| Q3 | 0.71 (0.5-1.01) | 0.053 |
| Q4 | 0.58 (0.4-0.84) | 0.004 |
| *P* for trend | 0.004 |  |
| Per SD increase | 0.85 (0.74-0.98) | 0.024 |
| Valine |  |  |
| Q1 | Reference |  |
| Q2 | 0.65 (0.46-0.92) | 0.014 |
| Q3 | 0.65 (0.46-0.91) | 0.013 |
| Q4 | 0.5 (0.35-0.72) | <0.001 |
| *P* for trend | < 0.001 |  |
| Per SD increase | 0.8 (0.69-0.92) | 0.002 |
| Total BCAA |  |  |
| Q1 | Reference |  |
| Q2 | 0.62 (0.44-0.87) | 0.006 |
| Q3 | 0.6 (0.43-0.85) | 0.004 |
| Q4 | 0.47 (0.33-0.68) | <0.001 |
| *P* for trend | < 0.001 |  |
| Per SD increase | 0.82 (0.71-0.94) | 0.005 |

BCAA: Branched-chain amino acid; GC: gastric cancer; HR: hazard ratio; CI: confidence interval; SD: standard deviation; Models were fully adjusted for age, sex, ethnicity, diet score, TDI, MET, BMI, smoking and drinking status, DM, Hypertension, CVD, lipid, and family history of cancer.

| **Table S5. The association between BCAAs and the risk of GC after multiple imputations of other four data sets** | | | | | | |
| --- | --- | --- | --- | --- | --- | --- |
| **Type** | Data set 1 | Data set 2 | Data set 3 | Data set 4 | Pooled results | *P* |
|  | HR (95% CI) | HR (95% CI) | HR (95% CI) | HR (95% CI) | HR (95% CI) |  |
| Isoleucine |  |  |  |  |  |  |
| Q1 | Reference | Reference | Reference | Reference | Reference |  |
| Q2 | 0.79 (0.59-1.07) | 0.79 (0.59-1.07) | 0.79 (0.59-1.07) | 0.79 (0.59-1.07) | 0.79 (0.59-1.06) | 0.121 |
| Q3 | 0.63 (0.46-0.86) | 0.63 (0.46-0.86) | 0.63 (0.46-0.86) | 0.63 (0.46-0.86) | 0.63 (0.46-0.86) | 0.004 |
| Q4 | 0.65 (0.48-0.89) | 0.66 (0.48-0.89) | 0.65 (0.48-0.89) | 0.65 (0.48-0.89) | 0.65 (0.48-0.89) | 0.007 |
| *P* for trend | 0.003 | 0.004 | 0.003 | 0.003 | 0.003 |  |
| Per SD increase | 0.86 (0.76-0.97) | 0.86 (0.76-0.97) | 0.86 (0.76-0.97) | 0.86 (0.76-0.97) | 0.86 (0.76-0.97) | 0.015 |
| Leucine |  |  |  |  |  |  |
| Q1 | Reference | Reference | Reference | Reference | Reference |  |
| Q2 | 0.69 (0.51-0.95) | 0.69 (0.51-0.95) | 0.69 (0.51-0.95) | 0.69 (0.51-0.95) | 0.69 (0.51-0.94) | 0.019 |
| Q3 | 0.74 (0.55-1) | 0.74 (0.55-1) | 0.74 (0.55-1) | 0.74 (0.55-1) | 0.74 (0.55-1) | 0.048 |
| Q4 | 0.57 (0.42-0.79) | 0.57 (0.42-0.79) | 0.58 (0.42-0.79) | 0.57 (0.42-0.79) | 0.57 (0.42-0.79) | 0.001 |
| *P* for trend | 0.002 | 0.002 | 0.002 | 0.002 | 0.002 |  |
| Per SD increase | 0.85 (0.75-0.96) | 0.85 (0.75-0.96) | 0.85 (0.75-0.96) | 0.85 (0.75-0.95) | 0.85 (0.75-0.96) | 0.009 |
| Valine |  |  |  |  |  |  |
| Q1 | Reference | Reference | Reference | Reference | Reference |  |
| Q2 | 0.66 (0.48-0.89) | 0.66 (0.48-0.89) | 0.66 (0.48-0.89) | 0.66 (0.48-0.89) | 0.66 (0.48-0.9) | 0.008 |
| Q3 | 0.65 (0.48-0.88) | 0.65 (0.48-0.88) | 0.65 (0.48-0.88) | 0.65 (0.48-0.88) | 0.65 (0.48-0.88) | 0.005 |
| Q4 | 0.53 (0.39-0.73) | 0.54 (0.39-0.74) | 0.53 (0.39-0.73) | 0.53 (0.39-0.73) | 0.53 (0.39-0.73) | <0.001 |
| *P* for trend | <0.001 | <0.001 | <0.001 | <0.001 | <0.001 |  |
| Per SD increase | 0.8 (0.71-0.91) | 0.81 (0.71-0.91) | 0.8 (0.71-0.91) | 0.8 (0.71-0.91) | 0.8 (0.71-0.91) | 0.001 |
| Total BCAA |  |  |  |  |  |  |
| Q1 | Reference | Reference | Reference | Reference | Reference |  |
| Q2 | 0.62 (0.45-0.84) | 0.62 (0.45-0.84) | 0.62 (0.45-0.84) | 0.62 (0.45-0.84) | 0.62 (0.45-0.85) | 0.003 |
| Q3 | 0.6 (0.44-0.81) | 0.6 (0.44-0.81) | 0.6 (0.44-0.81) | 0.6 (0.44-0.81) | 0.6 (0.44-0.81) | 0.001 |
| Q4 | 0.51 (0.37-0.7) | 0.51 (0.37-0.7) | 0.51 (0.37-0.7) | 0.51 (0.37-0.7) | 0.51 (0.37-0.7) | <0.001 |
| *P* for trend | <0.001 | <0.001 | <0.001 | <0.001 | <0.001 |  |
| Per SD increase | 0.82 (0.72-0.93) | 0.82 (0.72-0.93) | 0.82 (0.72-0.93) | 0.82 (0.72-0.93) | 0.82 (0.72-0.93) | 0.002 |

BCAA: Branched-chain amino acid; GC: gastric cancer; HR: hazard ratio; CI: confidence interval; SD: standard deviation; Models were fully adjusted for age, sex, ethnicity, diet score, TDI, MET, BMI, smoking and drinking status, DM, Hypertension, CVD, lipid, and family history of cancer.

| **Table S6. Fine-Gray proportional hazard regression models for the association between BCAAs and the risk of GC** | | |
| --- | --- | --- |
| Type | HR (95%CI) | *P* |
| Isoleucine |  |  |
| Q1 | Reference |  |
| Q2 | 0.8 (0.59-1.08) | 0.14 |
| Q3 | 0.63 (0.46-0.87) | 0.0047 |
| Q4 | 0.66 (0.48-0.9) | 0.009 |
| *P* for trend | 0.006 |  |
| Per SD increase | 0.86 (0.75-0.98) | 0.024 |
| Leucine |  |  |
| Q1 | Reference |  |
| Q2 | 0.7 (0.52-0.96) | 0.026 |
| Q3 | 0.76 (0.56-1.02) | 0.071 |
| Q4 | 0.59 (0.43-0.81) | 0.001 |
| *P* for trend | 0.004 |  |
| Per SD increase | 0.85 (0.75-0.97) | 0.019 |
| Valine |  |  |
| Q1 | Reference |  |
| Q2 | 0.67 (0.5-0.91) | 0.011 |
| Q3 | 0.65 (0.48-0.89) | 0.0065 |
| Q4 | 0.54 (0.4-0.75) | <0.001 |
| *P* for trend | < 0.001 |  |
| Per SD increase | 0.81 (0.71-0.93) | 0.0018 |
| Total BCAA |  |  |
| Q1 | Reference |  |
| Q2 | 0.62 (0.46-0.85) | 0.0025 |
| Q3 | 0.61 (0.45-0.83) | 0.0015 |
| Q4 | 0.52 (0.38-0.71) | <0.001 |
| *P* for trend | < 0.001 |  |
| Per SD increase | 0.83 (0.72-0.95) | 0.0054 |

BCAA: Branched-chain amino acid; GC: gastric cancer; HR: hazard ratio; CI: confidence interval; SD: standard deviation; Models were fully adjusted for age, sex, ethnicity, diet score, TDI, MET, BMI, smoking and drinking status, DM, Hypertension, CVD, lipid, and family history of cancer.
